# The Emerging Role of Neutrophils in the Pathogenesis of Thrombosis in COVID-19

**DOI:** 10.3390/ijms22105368

**Published:** 2021-05-20

**Authors:** Valeria Iliadi, Ina Konstantinidou, Konstantina Aftzoglou, Sergios Iliadis, Theocharis G. Konstantinidis, Christina Tsigalou

**Affiliations:** 1 Medical School, Izhevsk State Medical Academy, Kommunarov Street 281, 426034 Izhevsk, Russia; iliadi.valeria@mail.ru (V.I.); sergeyiliadis@mail.ru (S.I.); 2 Medical School, University of Patras, Rio, 26504 Patra, Greece; konstairene@yahoo.com; 3 Medical School, Comenius University, Spitalska 24, 81372 Bratislava, Slovakia; konnavtz@gmail.com; 4 Blood Transfusion Center, University General Hospital of Alexandroupolis Dragana Campus, 68100 Alexandroupolis, Greece; 5 Laboratory of Microbiology, Democritus University of Thrace, Dragana Campus, 68100 Alexandroupolis, Greece; xtsigalou@yahoo.gr

**Keywords:** COVID-19, SARS CoV-2, NETs, immunothrombosis

## Abstract

Previous studies have shown that COVID-19 leads to thrombotic complications, which have been associated with high morbidity and mortality rates. Neutrophils are the largest population of white blood cells and play a pivotal role in innate immunity. During an infection, neutrophils migrate from circulation to the infection site, contributing to killing pathogens. This mechanism is regulated by chemokines such as IL-8. Moreover, it was shown that neutrophils play an important role in thromboinflammation. Through a diverse repertoire of mechanisms, neutrophils, apart from directly killing pathogens, are able to activate the formation of thrombi. In COVID-19 patients, neutrophil activation promotes neutrophil extracellular trap (NET) formation, platelet aggregation, and cell damage. Furthermore, neutrophils participate in the pathogenesis of endothelitis. Overall, this review summarizes recent progress in research on the pathogenesis of COVID-19, highlighting the role of the prothrombotic action of neutrophils in NET formation.

## 1. Introduction

The novel coronavirus disease 2019 (COVID-19) was first reported in Wuhan, China, in December 2019 [1]. On 11 March 2020, the World Health Organization characterized the global health emergency of COVID-19 as a pandemic. As of 28 March 2021, 126,359,540 cases of COVID-19 have been confirmed, and 2,769,473 deaths have been reported globally [2]. The etiology of the disease is severe acute respiratory syndrome coronavirus 2 (SARS CoV-2), an enveloped, positive-sense RNA belonging to β-coronaviruses [3].

It was previously reported that elevated levels of blood neutrophils are an early indicator of SARS-CoV-2 severe infection [4]. Moreover, the elevation of different coagulation parameters, such as D-dimers, prothrombin time (PT), fibrinogen, and fibrinogen degradation products (FDPs), has been observed in patients with COVID-19 [5,6,7,8,9,10]. The coagulation mechanism includes specific proteins that act as natural anticoagulants, thereby avoiding formation of clots. These proteins are antithrombin (AT), protein C (PC), and protein S (PS). The results of previous studies, however, seem to be controversial. Zhang et al. reported that the activities of protein C, protein S, and antithrombin were all below the normal range [11]. Moreover, Gazzaruso et al. reported that COVID-19 patients have low levels of AT. The authors further suggested that AT is strongly associated with mortality in COVID-19 [12]. Antithrombin (AT) plays a significant role in COVID-19-induced coagulopathy, where low AT levels might explain the ineffectiveness of anticoagulants in patients with COVID-19 [13]. The above research highlights the synergistic roles of immune and coagulation systems in the development of thrombotic manifestations in COVID-19.

The mechanism of NETosis, its role in the pathogenesis of immunothrombosis and COVID-19-related coagulopathy, and therapeutic interventions targeting neutrophils are discussed in this review.

## 2. Neutrophils

Neutrophils are the “Cinderella” of innate immunity—except for their critical role in the host’s defense against pathogens—and play a vital role in thrombosis. Neutrophils are generated in the bone marrow and circulate in the blood as dominant white blood cells. Neutrophil migration is critical for host defense and pathogen clearance during infection [14]. The interactions between neutrophil adhesion receptors and adhesion molecules of the beta2 integrin family (CD11/CD18) on endothelial cells is necessary for neutrophil activation and migration to the site of infection [15].

Neutrophils’ antimicrobial arsenal is impressive and includes different effector mechanisms such as phagocytosis and degranulation. In 2004, Brinkmann et al. described a new additional antimicrobial paradigm of neutrophil action known as neutrophil extracellular traps (NETs) [16]. NETs are composed of chromatin and decorated with several proteins that have antimicrobial properties, such as histones, elastase, and myeloperoxidase [17]. Currently, it is widely accepted that there are three antibacterial mechanisms of neutrophil action: phagocytosis, degranulation, and NET formation. NETs exert their antimicrobial effects through pathogen immobilization via entrapment. Moreover, the NETs components of antimicrobial peptides, histones, and DNA produce direct antimicrobial effects [18,19,20]. Over the past decade, scientists have uncovered the vital role of neutrophils and neutrophil extracellular traps (NETs) in thrombo-inflammation [21,22,23,24].

## 3. Induction and Molecular Mechanisms of NET Formation

To date, a wide spectrum of stimuli has been recognized as inducers of NET formation (Table 1). Direct exposure to microbial pathogens, both Gram-positive bacteria (*Staphylococcus aureus, Staphylococcus suis, Streptococcus pyogenes, Streptococcus pneumoniae*) [8,9,10] and Gram-negative bacteria (*Escherichia coli, Salmonella enterica, Shigella flexneri, Pseudomonas aeruginosa)*, exposure to hyphae or yeast (*Candida albicans*), and exposure to protozoan parasites (*Leishmania amazonensis* or *Trypanosoma cruzi*) are able to induce NET generation [25,26,27]. Additionally, drugs such as statins and antibiotics can activate neutrophils to create NETs [19,28]. Jhunjhunwala S et al. further showed that sterile implant materials can induce NET formation [29].

As first shown by Zychlinsky et al., NET formation depends on the reactive oxygen species (ROS) produced by NADPH oxidase [17]. This process was confirmed in a study on patients suffering from chronic granulomatous disease (CGD) [30]. Fuchs et al. determined that mutations in nicotinamide adenine dinucleotide phosphate (NADPH) oxidase suspend NET generation. Interestingly, the results in this study demonstrated that the addition of exogenous ROS to CGD neutrophils promotes NET formation [30]. Similarly, Bianchi et al. showed that gene therapy in a patient with CGD restored NET formation through neutrophils, highlighting the role of functional NADPH oxidase in NET formation [31].

Papayannopoulos et al. further demonstrated the novel functions of granular proteins such as Neutrophil Elastase (NE) in the regulation of chromatin density [32] The authors demonstrated that activated NE escapes from azurophilic granules and translocates to the nucleus where it promotes chromatin decondensation through the degradation of specific histones. Consequently, myeloperoxidase synergizes with NE to drive chromatin decondensation, which contributes to NET formation. The decondensation of chromatin, an essential step of NET formation, is associated with the hypercitrullination of Histone H3 through the conversion of histone arginine to citrulline by peptidylarginine deiminase 4 (PAD 4), an enzyme that is particularly rich in mature neutrophils [33]. Hyperctrullination seems to play an essential role in NET formation. One study showed that mice deficient in PAD 4 were unable to form NETs and presented reduced NET-dependent bacterial trapping and killing [34].

Another mechanism involved in NET release is autophagy. Several studies have shown that autophagy modifies neutrophil functions [35,36]. Autophagy is a homeostatic mechanism involved in programmed cell death. Autophagic machinery is induced by PI3K hVPS34. It was demonstrated that the inhibition of PI3K with 3-methyladenine (3-MA), wortmannin, and LY294002 inhibits autophagy. Moreover, autophagy is negatively regulated by the protein kinase mammalian target of rapamycin (mTOR) [37].

Recently, Mazzoleni et al. reported that Panton–Valentine leukocidin (PVL) triggers an alternative NETosis process [38]. This study showed that PVL-induced NETs differ from NADPH oxidase-dependent NETosis and target the mitochondria.

## 4. Neutrophils, NETs, and Endothelial Damage

Endothelial cells (ECs) are a keystone player in the maintenance of normal hemostasis. The integrity of the vessel wall, in tandem with the expression of various inhibitors such as tissue factor pathway inhibitor (TFPI), thrombomodulin, protein C receptor, and heparin-like proteoglycans, manifests anticoagulative action [39]. Endothelial cell damage has also been detected in COVID-19 patients as a common feature of disease [40]. Recent publications suggest that COVID-19 affects other organs beyond the lungs, such as the heart and kidneys. Lindner et al. showed SARS-CoV-2 to be present in myocardial tissue during an autopsy [41]. One of the possible mechanisms of multiorgan damage was determined to be the endothelitis. COVID-19-related endothelitis induces systemic vascular endothelial dysfunction, which was observed in the disease’s complications [42]. Sh et al. hypothesized that endothelial cells could be activated by antibodies, NETs, and different circulating proteins, except under a direct viral effect [43]. Ackermann et al. performed autopsies on seven patients who died from COVID-19-associated or influenza-associated respiratory failure. The report showed that the incidence of thrombus formation in the pulmonary microvasculature was approximately nine times higher than that related to influenza (*p* < 0.001) [44]. In the same study, histological analysis of the lungs from patients who suffered influenza-associated respiratory failure showed diffuse alveolar damage with perivascular T-cell infiltration. Conversely, histological analysis of the pulmonary vessels in patients with COVID-19 revealed widespread thrombosis with microangiopathy [44]. At the beginning of 2021, Evert et al. reported the same results in their own autopsy findings. The autopsy revealed that patients with severe diffuse alveolar damage had developed endothelitis and capillaritis [45]. To correlate endothelial dysfunction with in-hospital mortality, Philippe et al. measured a panel of endothelial biomarkers and the von Willebrand factor (VWF) in 208 COVID-19 patients. According to the authors’ data, the best predictor for in-hospital mortality was VWF [46].

Skendros et al. investigated the role of the NET/platelet/thrombin axis in ECs and demonstrated that complement inhibition has a therapeutic effect in SARS-CoV-2 infection, which is reflected by a decline in C-reactive protein and IL-6 levels, marked improvements in lung function, and the resolution of SARS-CoV-2-associated acute respiratory distress syndrome (ARDS) [47].

## 5. Neutrophils, NETs, and Thromboinflammation

Apart from robust antimicrobial properties, through NETs, neutrophils also induce a vigorous procoagulant response. Activation of the coagulation system is a fundamental host defense mechanism that prevents the dissemination of infectious agents via fibrin deposition and thrombus formation. Although previous studies reported that neutrophils acquire but do not produce TF, which would attenuate the significance of neutrophils in thrombosis, it is generally accepted today that neutrophil-derived TF is involved in thrombosis [48]. Additionally, NET release has emerged as a major contributor to neutrophil-related thromboinflammation, providing the scaffold for platelet entrapment and subsequent activation. The leading role of NETs in neutrophil-related thromboinflammation was proven using both in vitro and ex vivo models, including in sepsis, deep venous thrombosis (DVT), and malignancies (Table 2) [49,50]. The presence of NETs was recently identified in thrombi in a murine model of deep vein thrombosis [49]. Brill et al. reported that NETs’ extracellular chromatin, which likely originates from neutrophils, is a structural component of a venous thrombus and that both the DNA scaffold and histones appear to contribute to the pathogenesis of DVT in mice. NETs may provide new targets for DVT drug development. Moreover, Kambas et al. demonstrated that the expression of bioactive TF in NETs can induce a coagulation cascade [51]. These authors shed light on the involvement of autophagic machinery in the expression of TF in NETs and the subsequent activation of thrombi formation.

In particular, in SARS Cov-2 patients, Leppkes et al. showed that severe disease induces NET formation inside the microvessels. The intravascular formation of NETs with platelet aggregation leads to organ damage due to rapid vessel occlusion [52]. Moreover, Nicolai et al. noted that inflammatory microvascular thrombi containing NETs and platelets can be found in the kidney, lung, and heart in COVID-19 patients [53].

Platelet–neutrophil cooperation is a noteworthy mechanism of innate immunity that contributes to the pathogenesis of thrombosis. After von Willebrand factor-dependent priming, platelets can affect neutrophils both through direct interactions and through the release of soluble mediators [54,55,56]. Platelets secrete a variety of different molecules. Inorganic polyphosphate (PolyP) is a critical component of platelet-dense granules that participates in coagulation and inflammation. The critical role of platelet-released PolyP was shown in the work of Morrissey et al. [57].

In addition, NET formation can also be induced by therapeutic manipulation. Extracorporeal membrane oxygenation (ECMO) therapy was used to improve the oxygenation of patients suffering from COVID-19. Ultimately, the mortality rates declined among patients who underwent ECMO. Nevertheless, thrombotic complications remained frequent. As foreign surfaces, the biomaterials of ECMO systems can induce NET formation in a platelet-independent manner [58]. To test this phenomenon, Winnersbach et al. studied the effect of ECMO on platelet functions and thrombotic complications. In this study, platelet-poor (PLT-) and naive (PLT+) heparinized human blood was circulated for 6 h in two identical in vitro test circuits used for ECMO devices. The authors reported that the depletion of PLTs within the ECMO system was associated with limited PLT activation but not sufficient to inhibit clot formation [59].

## 6. Neutrophils, NETs, and Lung Damage in COVID-19

The lung is the first organ that SARS-CoV-2 attacks. After arriving at the alveoli, SARS-CoV-2 triggers innate immune responses via the activation of alveolar macrophages. Then, viral particles activate a complement cascade via the lectin pathway. The complementary peptides C3a and C5a, produced as part of the activation of the complement system, stimulate the migration of neutrophils to the site of infection. The complement membrane attack complex (MAC) then exerts cell damage, thereby producing damage-associated molecular patterns (DAMPs). In addition, the SARS-CoV-2 S protein stimulates lung epithelial cells to release specific proteins such as epithelial membrane protein 2 (Emp2). The Emp2 of alveolar epithelial type 1 cells upregulates neutrophil migration. As part of the first line of innate immune response, the activated neutrophils perform protective roles in various infections through phagocytosis, degranulation, and NET formation. Furthermore, activated neutrophils, together with macrophages, are responsible for secreting proinflammatory cytokines such as IL-1b, IL-2R, IL-6, IL-8, TNF-α, and others [64,65,66]. Studies on lung histopathology in COVID-19 infection and severe respiratory failure have demonstrated the presence of distinct alveolar damage and thrombi formation in the peripheral pulmonary vessels. Interestingly, visualization of neutrophil-rich inflammation and neutrophil extra-cellular traps at the site of infection confirmed the role of neutrophils in COVID-19-related immune responses [67,68]. Veras et al. ascertained that SARS-CoV-2 can induce NET formation through healthy neutrophils, which depends on angiotensin-converting enzyme 2 or hypercitrullination [69]. Zuo et al. confirmed this result in an ex vivo study. In studying sera from COVID-19 patients, the authors detected higher NET markers (cell-free DNA, myeloperoxidase (MPO) DNA complexes, and/or citrullinated histone H3) [4]. Similarly, Ng et al. investigated NET markers in COVID-19 patients compared to healthy individuals, and all markers were found to be elevated. The authors concluded that NETs play a pivotal role in disease progression and thrombotic complications [70]. These results were also recently observed by Wang et al. in a whole-tissue transcriptomic analysis of lung tissue and bronchoalveolar lavage fluid (BALF). In this study, the most significantly up-regulated marker genes, both in the lung tissue and in BALF, were found in neutrophils (84 genes). Of these 84 genes, 16 genes were NET-associated genes. Among the NET-associated genes, gene promoters of proteinarginine deiminase type 4 (PAD4) activation and ROS-related genes were observed [71].

Pathological findings from autopsies of COVID-19 patients highlighted the key role of neutrophils in hyperinflammation [72,73,74]. Overall, neutrophil infiltration through Neutrophilic plugs was detected in COVID-19 patients via Neutrophil Elastase (NE), Myeloperoxidase (MPO), and Citrullinated Histone H3 (citH3) staining. Notably, these findings featured NETs and platelets [74]. On the other hand, Sinha et al. analyzed 39 patients suffering from ARDS due to COVID-19 and reported that ARDS was not associated with higher systemic inflammation [75]. Moreover, ARDS in this cohort was associated with a lower prevalence of the hyperinflammatory phenotype in comparison to matched patients recruited to the HARP-2 study (UK multicenter, randomized controlled trial of simvastatin) [75].

Notably, Calfee et al. reported that direct lung injury was correlated with severe lung epithelial injury, whereas an opposite pattern of predominantly endothelial injury was observed in indirect lung injury [76]. Based on these findings, the authors suggested different subtypes of ARDS due to COVID-19.

## 7. Neutrophils, NETs, and Kidney Damage in COVID-19 Thromboinflammation

Although the respiratory system is the first target of SARS-CoV-2, acute kidney injury (AKI) has also been described in patients suffering from COVID-19 [77]. The kidneys appear to be the second largest organ involved in COVID-19-related thromboinflammation. Cytokine storm, endothelial injury, and neutrophil extracellular trap release are some of the pathophysiological mechanisms that lead to renal capillary thrombosis during COVID-19 infections [78]. Clinical signs of kidney involvement include increased serum creatinine with or without new-onset proteinuria. Cheng et al. reported that kidney disease is associated with a high mortality index in patients with COVID-19 [79]. A kidney histopathological analysis of patients with COVID-19 confirmed the presence of virus-related lesions such as vasculitis, inflammation, and platelets with erythrocyte aggregates obstructing the lumen of the capillaries. In addition, an electron microscopic examination showed coronavirus-like particles in the tubular epithelium and podocytes. An immunostaining analysis revealed that ACE2 receptors were upregulated in patients with COVID-19 and colocalized with the SARS-CoV-2 nucleoprotein. Factors such as systemic hypoxia, abnormal coagulation, and possible drug or hyperventilation-relevant rhabdomyolysis contribute to acute kidney injury [80]. Furthermore, activation of the complement system might be involved in renal injury in COVID-19. Using immunohistochemistry for the complement factors C1q, MASP-2, C3b, C3d, C4d, and C5b-9, Pfister et al. investigated the involvement of the complement system in renal injury in six kidney biopsies from the autopsy materials of patients with COVID-19 [81]. Both C3 cleavage products (C3b and C3d) were detected in the renal arteries and glomerular capillaries of the COVID-19 biopsies. The membrane attack complex C5b-9 (MAC) was predominantly deposited in the peritubular capillaries, renal arterioles, and tubular basement membrane.

## 8. Neutrophils, NETs, and Kawasaki Disease

Kawasaki disease (KD) is an acute febrile systemic vasculitis in small and medium-sized arteries that leads to coronary artery lesions (CALs) in young children, especially in Japan [82,83]. Since Kawasaki disease exhibits seasonal and regional patterns, it was suggested that other infections may be a trigger for KD. Indeed, coronavirus HCoV-229E was identified as the etiological factor in the development of KD [84]. Since KD diagnosis is only based on clinical criteria, commonly used biochemical markers such as CRP cannot distinguish KD from infectious diseases. In this context, a better understanding of the pathophysiological mechanisms of KD could help discover new biomarkers for the early detection of KD.

Histopathologic studies of KD vasculitis lesions have demonstrated that the predominant cells are CD163 monocytes/macrophages and CD3 T cells [85]. Other inflammatory cells, such as neutrophils, and neutrophil-driven proinflammatory cytokine production also contribute to EC damage. Armaroli et al. reported that EC damage with elevated serum S100A12 levels in patients with KD was strictly dependent on interleukin-1β (IL-1β) signaling (*p* < 0.001) [86]. Although it was previously shown that SARS-CoV-2 infection is a type of NETopathy, Yamashita et al. reported for the first time that serum from KD patients can stimulate NET formation in human neutrophils in vitro [87]. This study demonstrated that NET formation is the principal pathophysiological mechanism in SARS-CoV-2 infection and Kawasaki-like syndrome [87]. This result agrees with a study by Pouletty et al. that indicated Kawasaki-like syndrome to be associated with SARS-CoV-2 infection in children [88]. Furthermore, Jing et al. analyzed the role of neutrophil extracellular traps (NETs) in the pathogenesis of KD [89]. The authors found that the neutrophils of KD patients induced NET formation and that those NETs significantly increased pro-inflammatory cytokine production and NF-κB activation in peripheral blood mononuclear cells (PBMCs). In addition, in vitro models in an endothelial cell culture showed the increased expression of vascular endothelial growth factor A (VEGF-A) and hypoxia-inducible factor-1α (HIF-1α). These results demonstrate that NETs are key players in the pathogenesis of KD and Kawasaki-like syndrome.

## 9. Neutrophils, NETs, and Therapeutical Interventions in COVID-19

It is well-documented that neutrophils are key factors in the cytokine storm and thrombotic complications experienced by COVID-19 patients and that therapeutic targeting of neutrophils might ameliorate hyperinflammatory syndrome in COVID-19. Therefore, several studies proposed that NETs could be a therapeutic target in COVID-19 patients (Table 3) [90,91,92,93].

### 9.1. Interleukin Targeting

The cytokines involved in the pathogenesis of lung inflammation in COVID-19 include IL-1, IL-6, IL-8, and TNF-α [94]. During the early phases of the pandemic, off-label administration of an FDA-approved anti-interleukin drug was reported. However, the results of related studies are conflicting.

Several studies of COVID-19 patients reported positive effects of interleukin-1 antagonists [95,96]. However, the targeting of IL-1 receptors by anakinra (an IL-1 receptor antagonist (Ra)) in COVID-19 remains uncertain. To date, there are three FDA-approved anti IL-1 agents: Anakinra is an IL-1 receptor inhibitor, while canakinumab and rilonacept are IL-1β inhibitors. Anakinra was reported in the treatment of COVID-19. The administration of anakinra to COVID-19 patients was found to be safe and might be associated with a reduction in both mortality and the need for mechanical ventilation [96]. On the other hand, the CORIMUNO-ANA-1 study reported that Anakinra did not improve outcomes in patients with mild-to-moderate COVID-19 pneumonia [97]. Similarly, a single-center retrospective comparative study was performed by de la Calle et al., which reported that treatment with anakinra was not able to improve the prognosis of patients with tocilizumab-refractory severe COVID-19 [98].

Several ongoing clinical studies are using anti-IL-6 in therapeutical protocols for COVID-19. Guaraldi et al. performed a study on adult patients to assess the role of anti-IL-6 in reducing the risk of death in patients with severe COVID-19. This observational cohort study included 544 severe COVID-19 patients; 365 of these patients received standard protocols, while the remaining 179 patients were treated with tocilizumab (a monoclonal antibody that binds to the IL-6 receptor). The results of this study showed that treatment with tocilizumab reduced the risk of invasive mechanical ventilation or death in patients with severe COVID-19 pneumonia [99]. The same results were reported by Huang et al., showing that tocilizumab treatment is associated with a reduction in death compared to non-treatment in severe COVID-19 patients [100]. In contrast with previously reported studies, Stone et al. recently reported that Tocilizumab was not effective in preventing intubation or death for moderately ill patients hospitalized with COVID-19 [101].

Interleukin-17 was previously related to a hyperinflammatory state in COVID-19 patients. The role of anti-IL-17 was reported in a patient with ankylosing spondylitis treated with secukinumab (a monoclonal antibody that binds to the IL-17 protein). According to this study, IL-17 inhibitors were presented as promising targets for the prevention of aberrant inflammation and acute respiratory distress in COVID-19. Positive results from anti-IL-17 were also reported by Mareev et al. [102]. Mugheddu et al. further reported that two COVID-19-positive patients who also suffered from psoriasis recovered rapidly from their infections as a result of a long-term secukinumab treatment [103].

Overall, a variety of terminated and ongoing studies focused on IL receptors. Despite the promising results from some of these studies, other research has provided opposite conclusions, leading to a debate surrounding the true value of these interventions. Better-designed and multicenter studies could elucidate the true value of these drugs.

### 9.2. Neutrophil Elastase Inhibitors

Neutrophil Elastase (NE), a serine protease, is one of the proteolytic enzymes that contribute greatly to the functions of neutrophils. NE participates in neutrophil activation and NET formation. Specifically, NE facilitates the invasion of SARS-CoV-2 into host cells and can also damage lung tissues directly. The role of NE in patients with ARDS, including COVID-19, and sepsis have been reported in different studies [104,105,106,107]. Moreover, in an experimental mice model, Ogura et al. reported that NE deficiency improves myocardial injury in post-myocardial infarction [108]. Based on previous reports, NE inhibition could potentially have positive effects on COVID-19 [109]. On the other hand, the results from a STRIVE study on a total of 492 mechanically ventilated heterogeneous acute-lung-injury patients showed intravenous administration of the NE inhibitor sivelestat to have no effect on 28-day all-cause mortality or ventilator-free days [110].

### 9.3. DNase Inhibitors

Experimental studies showed that the application of DNase I in a therapeutic scheme for acute lung injury due to severe bacterial pneumonia improved the survival rates of mice via a reduction in NET formation [111,112]. Moreover, treatment with DNase increased survival in cystic fibrosis (CF) patients. CF is characterized by the presentation of abundant extracellular DNA (eDNA) in the airways [113]. Recently, Weber et al. reported data on the administration of Dornase alfa, recombinant human DNase-1, for the treatment of five mechanically ventilated patients with COVID-19. The results suggested that Dornase alfa was well-tolerated by the patients [114]. Based on this case study, there are now several COVID-19 clinical trials using Dornase alfa. Desilles et al. published a structured summary of a study protocol for a randomized controlled trial on the efficacy and safety of aerosolized intra-tracheal Dornase alfa administration among patients with SARS-CoV-2-induced acute respiratory distress syndrome (ARDS) [115].

### 9.4. Colchicine

Colchicine is an alkaloid extract from the Autumn crocus that has been used to treat several inflammatory diseases, such as gout and familial Mediterranean fever (FMF), for many years [116,117]. Colchicine is one of the most ancient herbal remedies used for joint pain. To date, basic and clinical studies suggest that colchicine offers cardiovascular benefits [118]. The athero-protective potential of colchicine is based on its effects on tubulin-colchicine-complex polymerization, as well as its ability to suppress proinflammatory cytokine (IL-1β and IL-18) release by interacting with the Nod-like receptor protein 3 inflammasome protein complex [118,119]. Moreover, colchicine inhibits NET formation in patients with Acute Coronary Syndrome (ACS) [120]. Based on its effects, colchicine was proposed for the prophylaxis of venous thromboembolism in patients with COVID-19 [121,122]. Recently, the effects of colchicine on cardiac and inflammatory biomarkers were reported in the GRECCO-19 randomized clinical trial. The results showed that participants who received colchicine had statistically significantly improved time to clinical deterioration [123]. Scarsi et al. further supported the use of colchicine for the treatment of COVID-19 [124]. Furthermore, in a randomized, double-blinded, placebo-controlled clinical trial, Lope et al. reported that colchicine reduced the length of both supplemental oxygen therapy and hospitalization [125]. This study confirmed the results of previous research. The repurposing of colchicine or other drugs may, therefore, be helpful in the battle against COVID-19. However, further research needs to be evaluated and tested in upcoming clinical trials.

### 9.5. Corticosteroids

In the past, corticosteroids, as down-regulators of systemic inflammation, were administered to critically ill patients with conflicting results [126,127]. Recently, based on data from the RECOVERY trial (a large, multicenter, randomized, open-label trial performed in the United Kingdom), corticosteroids have been recommended for COVID-19 patients. In this trial, a total of 2104 patients started dexamethasone, and 4321 continued the usual therapy. This trial showed the mortality at 28 days to be lower among patients who were randomized to receive dexamethasone [128]. Moreover, the CoDEX Randomized Clinical Trial showed that Dexamethasone might attenuate lung injury in COVID-19 patients [129]. Among patients with moderate or severe COVID-19, the use of intravenous dexamethasone increased the number of ventilator-free days. Despite the positive results from the largest trials, RECOVERY and CoDEX, the benefitrisk factors for all COVID-19 patients must be estimated before corticosteroid treatment is started. Corticosteroid therapy in combination with cytokine storm is associated with significant insulin resistance and reduced insulin production from the pancreatic β cells. This dual hit can lead to severe hyperglycaemia and life-threatening complications. Future studies on the usefulness of corticosteroids in COVID-19 could provide insights into their beneficial and harmful effects.

### 9.6. Other Therapeutic Intervention That Affects Neutrophils

Prebiotics and probiotics could be considered potential preventive or therapeutic interventions to extenuate COVID-19 [130]. It was previously reported that some microorganisms, such as *Lactobacillus rhamnosus*, accept the activities of probiotics and reduce the functional activity of neutrophils both by inhibiting NET formation and by downregulating phagocytic activity [131]. Based on the well-documented role of NETs in the pathogenesis of COVID-19, the inhibition of NETs by *Lactobacillus rhamnosus* may exert a positive effect on COVID-19 patients. Moreover, the microbiota disruption (dysbiosis) during the COVID-19 pandemic has led to increasing incidences of *C. difficile* infection (CDI) [132]. Probiotics have been further reported to promote immunity to secondary infections such as pseudomembranous colitis due to *Clostridium difficile*. Overall, nutraceuticals could be a hidden weapon to target SARS-CoV-2.

## 10. Conclusions

**Figure 1:**
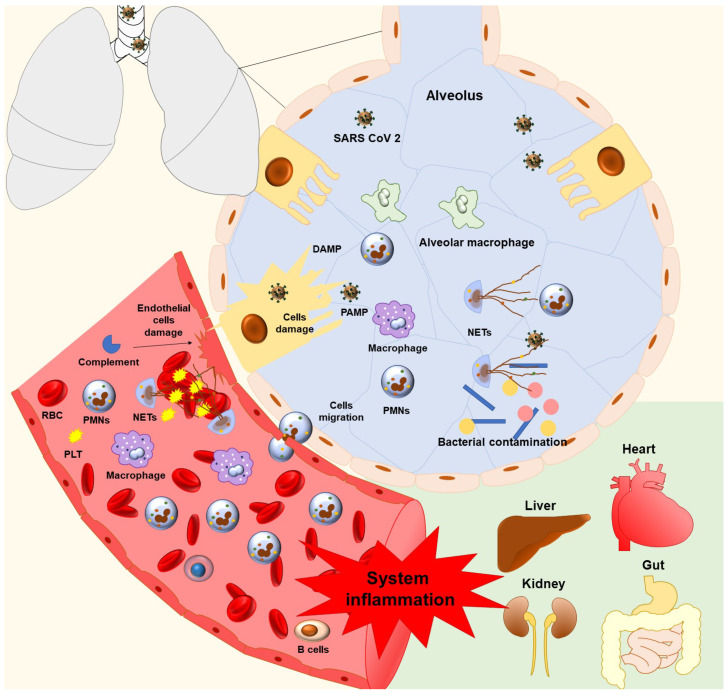
Neutrophil and thromboinflammation in COVID-19. Host–virus interplay includes virus-related alveolar macrophage activation, which leads to a cascade of inflammatory stimuli, cell activation, and migration. Signals from different chemokines induce neutrophil–endothelium interactions, allowing neutrophil to crawl and arrest along the endothelium. Neutrophils accomplish their extravasation predominantly via endothelial cell–cell junctions. At the site of infection, neutrophils activate phagocytosis, degranulation, and NET formation, which can protect the host during its virus response. On the other hand, neutrophils can exacerbate lung hyperinflammation and cytokine storm in COVID-19 patients. Ultimately, NETs interact with the platelets and complements, thereby causing endothelial cell damage and triggering thrombi formation. Hyperinflammation and Inflammatory microvascular thrombi then lead to system inflammation, which affects kidney, gut, liver, and heart function. Abbreviations: SARS-CoV-2: severe acute respiratory syndrome coronavirus 2; PAMP: pathogen-associated molecular pattern; DAMP: danger-associated molecular pattern; NETs: neutrophil extracellular traps.

Previous studies on the pathophysiological mechanisms of SARS-CoV-2 indicate that the innate immune system is an essential mechanism for the induction of COVID-19. Neutrophils, the “Cinderella” of innate immunity, participate in COVID-19 disease (Figure 1). NETs were described for the first time in 2004 by Brinkmann et al. and have often been studied under severe inflammatory conditions. The pathogenesis of COVID-19 includes both septic and aseptic inflammation, and viral infection triggers immune hyperactivation. Subsequently, this hyperactivation leads to cytokine storm and has been associated with COVID-19 complications such as ARDS, multiorgan dysfunction, and thromboembolic phenomena. Finally, therapeutic interventions targeting neutrophils may represent a potential component in an integrated therapeutic strategy for COVID-19 patients.

## Figures and Tables

**Table 1:** NET inducers. tnterleukin 8 (IL-8), tumor necrosis factor -α (TNFα), interferon-γ (IFN-γ), interferon-α (IFN-α), granulocyte-macrophage colony-stimulating factor (GM-CSF), lipopolysaccharides (LPS), and complement component 5a (C5a).

Microorganisms	Cytokines/Chemokines
Bacteria	IL-8
*Escherichia coli*	TNFα
*Enterococcus faecalis*	IFN-γ
*Haemophilus influenzae*	IFN-α
*Shigella flexneri*	GM-CSF
*Staphylococcus aureus*	GM-CSF + LPS
*Streptococcus pyogenes*	C5a
*Streptococcus pneumoniae*	**Other inducers**
*Serratia marcescens*	Activated Platelets
*Pseudomonas aeruginosa*	Drugs
Intracellular bacteria	Statins
*Listeria monocytogenes*	Antibiotics
*Mycobacterium tuberculosis*	MSU monosodium urate crystals
Fungi yeast	PMA
*Aspergillus fumigatus*	Sterile Implant Materials
*Candida albicans*	
*Cryptococcus neoformans*	
Parasites	
*Toxoplasma gondii* *Leismania amazonensis*	
*Trypanosoma cruzi*	
Viruses	
HIV-1	
RSV—(respiratory syncytial virus)	
Influenza A	
SARS-CoV-2	

**Table 2:** Studies of NETs in thromboinflammation. Myeloperoxidase, MPO; Neutrophil Elastase, NE; High Mobility Group Box 1, HMGB-1; Deoxyribonucleic Acid, DNA; ST-segment elevation myocardial infarction, STEMI; Interleukin-1b, IL-1b; Interleukin-17, IL-17; Western blot, WB; Immunohistochemistry staining, IHC; systemic lupus erythematosus, SLE; Regulated in development and DNA damage responses 1, REDD-1; and Deep Vein Thrombosis, DVT.

Model	Target		Authors
	Proteins	NET Detection Method	
DVT (Animal)	MPO, TF	Immunofluorescence ELISA	Bril et al. [49]
STEMY (Human)	MPO, NE, TF	Immunofluorescence WB, ELISA	Stakos et al. [60]
Sepsis (Human)	MPO, NE, TF	Immunofluorescence ELISA	Kambas et al. [51]
Ischemic stroke (Animal)	Cit H3 HMGB-1	Immunofluorescence WB	Kim et al. [61]
COVID-19 (Human)	Cit H3	IHC, Immunofluorescence, WB	Leppkes et al. [52]
Ischemic stroke (Human)	Cit H3, MPO, NE, TF	Immunofluorescence ELISA	Zhou et al. [62]
SLE (Human)	REDD-1, MPO, NE, TF	Immunofluorescence ELISA	Frangou et al. [63]

**Table 3:** Therapeutic targeting of Neutrophil Extracellular Traps. Peptidylarginine deiminases -4, PAD-4; Neutrophil Elastase, NE; High Mobility Group Box 1, HMGB-1; Deoxyribonucleic Acid, DNA; Interleukin-1b, IL-1b, and Interleukin-17, IL-17.

Mechanisms	Target		Drug
	Proteins	Action	
Inhibition of NET formation	PAD-4	Inhibition of histone citrullination	PAD-4 inhibitor
	NE	Inhibition of proteas activity	Sivelestat
	NF-κB	NF-κB signaling pathway inhibition	Aspirin
	HMGB-1	HMGB-1-targeting	HMGB-1 inhibitors
NETs dissolution	DNA	NET degradation	DNase Dornase alfa
	DNA–Histone complex	NET degradation	Heparin
NETs protein blocking	IL-1b	IL-1b receptor antagonist	Anakinra
		Anti-IL-1b Abs	Canakinumab
		Inhibition of IL-1b secretion	Colchicine
	IL-17	Anti-IL-17 Abs	Secukinumab

## Data Availability

Not applicable.
